# Challenges and strategies associated with CAR-T cell therapy in blood malignancies

**DOI:** 10.1186/s40164-024-00490-x

**Published:** 2024-02-24

**Authors:** Zhaoyun Liu, Wenhui Lei, Hao Wang, Xiaohan Liu, Rong Fu

**Affiliations:** 1 Department of Hematology, Tianjin Medical University General Hospital, 154 Anshan Street, Heping District, Tianjin, 300052 PR China; 2 Tianjin Key Laboratory of Bone Marrow Failure and Malignant Hemopoietic Clone46Control, Tianjin, 300052 P. R. China; 3 Department of Nephrology, Lishui Municipal Central Hospital, Lishui, Zhejiang 323000 People’s Republic of China

**Keywords:** CAR-T cell therapy, Hematologic malignancies, Resistance and relapse mechanisms

## Abstract

Cellular immunotherapy, particularly CAR-T cells, has shown potential in the improvement of outcomes in patients with refractory and recurrent malignancies of the blood. However, achieving sustainable long-term complete remission for blood cancer remains a challenge, with resistance and relapse being expected outcomes for many patients. Although many studies have attempted to clarify the mechanisms of CAR-T cell therapy failure, the mechanism remains unclear. In this article, we discuss and describe the current state of knowledge regarding these factors, which include elements that influence the CAR-T cell, cancer cells as a whole, and the microenvironment surrounding the tumor. In addition, we propose prospective approaches to overcome these obstacles in an effort to decrease recurrence rates and extend patient survival subsequent to CAR-T cell therapy.

## Introduction

The application of cellular immunotherapy, precisely that which employs chimeric antigen receptor T (CAR-T) cells, has yielded substantial advancements in the management of malignant tumors, with a particular focus on blood cancers. It is worth noting that targeted CD19-CAR-T cell therapy has demonstrated efficacy in the treatment of aggressive and treatment-resistant acute leukemia [1]. As a result of the positive outcomes observed in clinical trials (Table 1), numerous cell products are now extensively employed in clinical practice [2–11].

**Table 1:** Clinical data of CAR-T cell products in hematologic malignancies

Products	Target Antigen	Indications	Patients (n)	ORR/CR	PFS/OS	MFU,	Reference
Ciltacabtagene autoleucel	BCMA	R/R MM	R/R MM patients(97)	97.9%/82.5%	54.9%and 70.4%(27mo)	28 mo	2
Idecabtagene vicleucel	BCMA	R/R MM	R/R MM patients(128)	73%/33%	27.3%/64%(12mo)	13.3 mo	3
Lisocabtagene maraleucel	CD19	R/R large B-cell lymphoma	R/R large B-cell lymphoma (269)	73%/53%	44%/58%(12 mo)	18.8mo	4
Brexucabtagene autoleucel	CD19	R/R MCL	R/R MCL(33)	91%/79%	51%(12mo)/ 61% (12MO)	-	5
Brexucabtagene autoleucel	CD19	R/R B-cell precursor ALL	R/R B-cell precursor ALL (65)	71% /56%	12.8mo/18.2	16.4mo	6
Axicabtagene ciloleucel	CD19	R/R large B-cell lymphoma	R/R large B-cell lymphoma(180)	83%/65%	41%/61%(24mo)	24.9	7
Axicabtagene ciloleucel	CD19	R/R follicular lymphoma	follicular lymphoma(124) and marginal zone lymphoma(24)	92%/74%		17.5	8
Tisagenlecleucel	CD19	R/R B-cell precursor ALL	r/r B-cell ALL(75)	81%/60%	90%/82%(6mo)	13.1	9
Tisagenlecleucel	CD19	R/R large B-cell lymphoma	R/R diffuse large B-cell lymphoma(93)	52%/40%	83%/49%(12mo)	14	10

ORR-overall response rate; CR: complete remission; PFS: progression-free survival; OS: overall survival; MFU: median follow-up;mo:months

Kymriah® (Tisagenlecleucel) has been extensively utilized to treat relapsed or refractory B-cell Acute Lymphoblastic Leukemia (ALL), demonstrating high rates of complete remission and manageable toxicity in the ELIANA trial [12]. The efficacy of the treatment for Diffuse Large B-cell lymphoma (DLBCL) was validated in the JULIET trial, resulting in its approval [10]. Additionally, Breyanzi® (Lisocabtagene maraleucel) received approval for r/r LBCL after the successful TRANSCEND trial [4]. Furthermore, the approval of Abecma® (Idecabtagene vicleucel) for the treatment of recurrent multiple myeloma (MM) was obtained due to the favorable results reported from the KarMMa trial [13].

Although CAR-T cell treatment has demonstrated substantial advancements in enhancing outcomes for r/r blood malignancies, attaining enduring and viable full remission for blood malignancy continues to pose a difficulty. The therapeutic efficacy of CAR-T cells is influenced by factors such as limited in vivo proliferation, B cell aplasia, tumor invasion, and treatment-related toxicities, including off-target effects [14–17]. An in-depth understanding of the fundamental factors behind treatment failure is necessary to overcome the obstacles presented by the limitations of current therapeutic approaches. Differentiating between intrinsically low T cell functioning and CAR-T cell malfunction in vivo is crucial. Numerous methodologies and strategies have been suggested for addressing these concerns, encompassing the enhancement of CAR-T cell structure and alterations of target antigens. Furthermore, there have been suggestions to combine with clinically approved medications such as chemotherapeutic treatments, monoclonal antibodies, or small molecule inhibitors.

This study examines the processes that contribute to the problems associated with resistance to CAR-T cell therapy, as well as the tactics that can be used to overcome resistance and recurrence in hematological malignancies.

## CAR structure

**Fig. 1:**
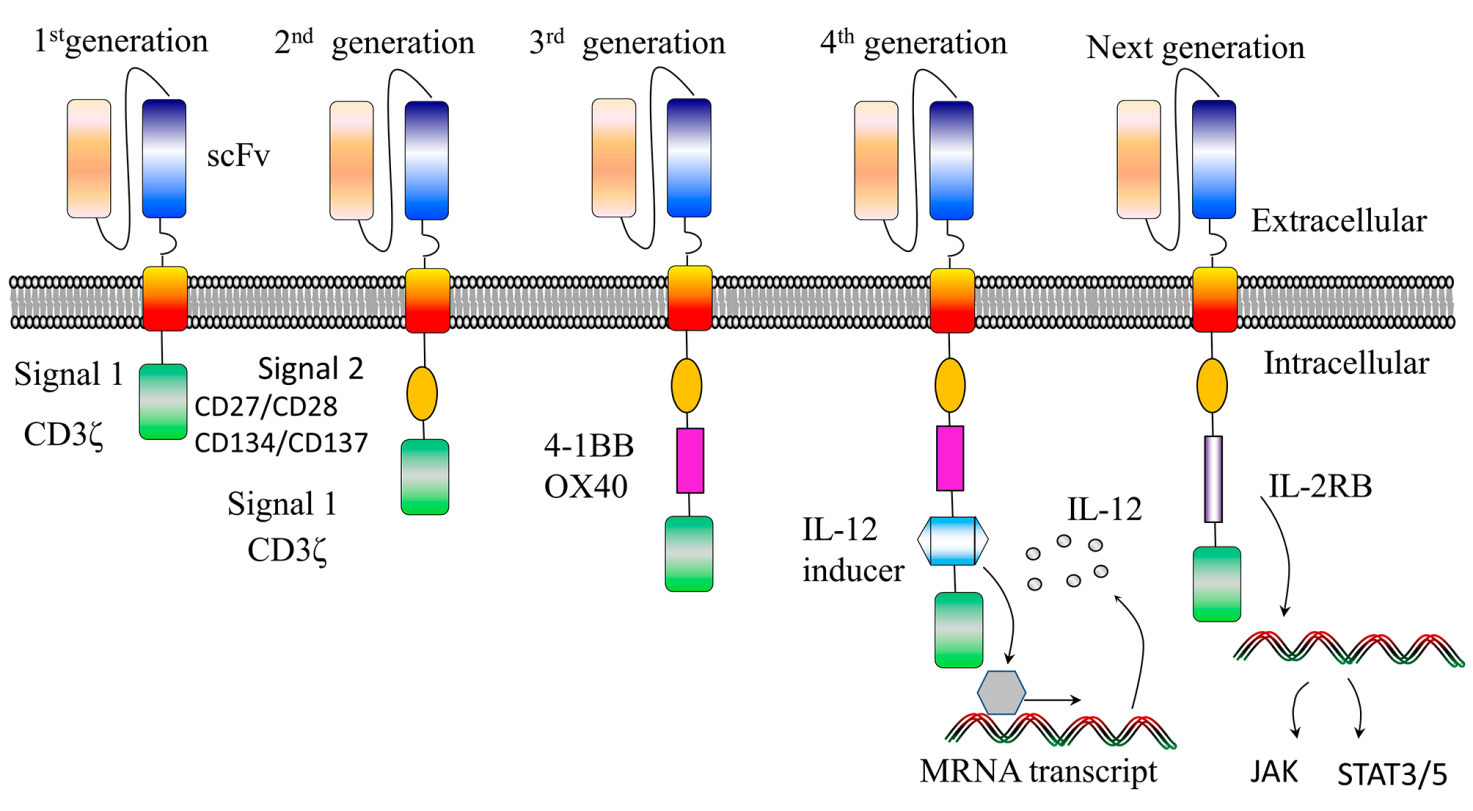
CAR structure and generations. CAR T cells are composed of three functional regions from the inside out: (1) the signaling domain of the TCR, (2) the transmembrane domain, and (3) the scFv. 1st generation: CD3ζ as signal transduction domain;2nd generation: additional costimulatory signaling domains (CD28 or 4-1BB);3rd generation: both CD28 and 4-1BB as co-stimulatory domains;4th generation: genes added for cytokine expression; Next generation: IL-2R β fragment added to 2nd generation CARs, inducing JAKs and STAT-3/5 production by mRNA transcription

The CAR is comprised of four primary structural elements: a cytoplasmic signaling domain, a hinge, a transmembrane domain, and an extracellular antigen-recognition domain (Fig. 1) [18]. Each domain has specific functions, and modifying the structures of the domains allows for different functionalities of the CAR.

### Extracellular antigen-recognition domain

This domain is responsible for recognizing and binding to target antigens. Typically, the antigen-binding region consists of variable light (VL) and variable heavy (VH) chains from monoclonal antibodies. The chains are interconnected through flexible linkers in the middle, forming single-chain variable fragments (scFv) [19, 20]. Although initially designed to specifically target and attach to tumor-specific antigens located outside of cells, subsequent research has demonstrated that the scFv also possesses the capability to identify tumor-specific antigens found inside cells [21, 22].

The structure, location, and interaction mode of the VH and VL chains influence both CAR specificity and affinity [23], with scFv affinity significantly affecting the anti-tumor properties. A high affinity for antigen binding is beneficial for the efficient identification of tumor-associated antigens (TAAs), which in turn triggers signal transduction and T-cell activation. Excessive affinity, however, can result in cytotoxicity or toxicity in T cells harboring CAR due to their interaction [24–26]. Interestingly, scFvs that target the same protein with similar affinity may have varying effects on the anti-tumor capabilities of CAR-T cells [27]. Therefore, optimizing the pairing between CAR and its target is crucial for identifying the ideal scFv for CAR engineering.

Additionally, certain scFvs can trigger ligand-independent tonic signaling and induce the differentiation and even death of effector T cells. This may impair CAR-T-cell proliferation, ultimately leading to disease recurrence [28]. Therefore, when developing CARs, it is of utmost importance to select scFvs that prevent the initiation of ligand-independent tonic signaling and to thoroughly evaluate aspects such as the density of the target antigen and the position of the epitope [29].

### Hinge and transmembrane domains

These domains have crucial functions in enabling access to specific antigens and controlling the activation of CAR-T cells. The hinge region serves as a linkage between the extracellular region, where the antigen attaches, and the cytoplasmic domain, which is responsible for transmitting signals. This linkage provides the necessary flexibility and length to overcome obstacles caused by steric hindrance. Significantly, the characteristics of the hinge can impact various aspects of CAR function, including flexibility, epitope recognition, activation output strength, and CAR expression [30, 31].

Cytokine release by CAR-T cells is also affected by the unique characteristics of the hinge and transmembrane domains. In vivo studies have suggested that T cells with CD28 domains exhibit inferior anti-tumor effects relative to those with CD8 domains [32]. Furthermore, these domains may have distinct functions, with the hinge regulating the CAR signal threshold. In contrast, the transmembrane domain controls the intensity of the signal by regulating the CAR expression level [33]. CAR-T cells having domains derived from CD8αhave been found to enhance CAR expression and induce tonic signaling, resulting in high-level secretion of IL-2, ultimately leading to increased cell activation [34]. Furthermore, T cells produced with CD19-CAR and including CD28 and CD8 transmembrane domains have different activities. The former interacts with natural binding partners to control the activity of CAR-T cells [35].

### Cytoplasmic domain

The intracellular domain of CAR usually contains at least one co-stimulatory domain together with an activation domain (Fig. 1). Optimizing the co-stimulatory functions is a vital factor to consider in CAR engineering in order to produce an ideal domain structure.

Early attempts in CAR-T cell development used complexes between immunoglobulin scFvs and CD3, which were first described in 1989 [18]. However, the tyrosine-based activation motifs did not activate T lymphocytes effectively, resulting in limited cytokine secretion and reduced in vivo survival time [36]. Clinical studies also showed low efficacy, confirming these findings [37].

Subsequent generations of CARs included co-stimulatory molecules, such as CD137 (4-1BB), CD134, and CD28, in the chimeric receptor. This inclusion enabled T cells to achieve sustained proliferation both in vitro and in vivo, along with robust cytokine secretion, leading to high response rates in clinical or preclinical studies [38, 39]. Among these co-stimulatory domains, CD28 and 4-1BB have received significant research attention and obtained FDA approval for their incorporation into CAR-T cell products [40]. Novel co-stimulatory domains, such as CD27 and OX40 (CD134), have been found to be effective, although they have not yet been examined in clinical settings [41–43].

Recent advancements in fourth-generation CAR-T cell therapies have enabled the targeted production of specific cytokines, such as IL-12, which can attract and activate other immune cells [44]. Utilizing proliferative cytokines such as IL-2 and IL-15 has demonstrated the ability to enhance the effectiveness of CAR-T cells, resulting in robust anti-tumor activity [45]. IL-15 plays a crucial role in maintaining the balance and survival of T cells. Research has shown that CD19 CAR-T cells, which have IL-15 expression driven by the antigen, exhibited enhanced survival, growth, and effectiveness against B cell malignancies in animal models. In addition, the potential of IL-7 and IL-21 to augment the effectiveness and durability of CAR-T cells has also been examined [46]. Studies have demonstrated that IL-7 facilitates the balanced growth of T cells, whilst overexpression of IL-21 has been found to sustain the long-term presence of T cells in vivo. Research has shown that the continuous production of IL-7 in CD19-CAR-T cells improves the effectiveness of fighting against tumors. On the other hand, the expression of IL-21 in CAR-T cells has demonstrated significant growth and long-lasting presence in xenograft models, leading to enhanced tumor regulation and increased survival [47]. These data strongly support the requirement to assess further the effectiveness of CAR-T cells that simultaneously express IL-15 and IL-21 in cancer patients.

Moreover, the failure of CAR-T cells to recognize antigen-negative tumor cells can contribute to tumor recurrence after treatment. Nevertheless, fourth-generation cells can resolve this issue by releasing targeted cytokines, resulting in improved T-cell activity. In addition, they attract and activate innate immune cells, leading to the efficient elimination of antigen-negative tumor cells in specific areas [48].

## Challenges and opportunities associated with CAR-T cell treatment of hematological cancers

CAR-T cell treatment is recognized as a significant option for treating various cancers, especially blood malignancies. However, clinical trials have encountered various difficulties, such as disease relapse and refractory cases. In this section, as shown in Tables 2 and 3, the state of CAR-T cell application for the treatment of hematological malignancies is evaluated, along with its challenges.

**Table 2:** Ongoing CAR-T cell therapy trials for hematologic malignancies

Clinical Study	Study Type	Indication	Target Antigen	Status	Sponsor
NCT04340154	Phase II	ALL	CD19 and CD22	Recruiting	Beijing Boren Hospital
NCT05727683	Phase I	R/R B-ALL	CD19	Recruiting	Shanghai Ming Ju Biotechnology Co., Ltd.
NCT05381662	phase I/II	ALL	CD19	Recruiting	Shanghai Unicar-Therapy Bio-medicine Technology Co.,Ltd
NCT04840875	Phase I	Acute T-cell leukemia / lymphoma	CD7	Recruiting	Beijing Boren Hospital
NCT04340167	phase II	R/R B-ALL	CD22	Recruiting	Beijing Boren Hospital
NCT04778579	phase II	R/R B-ALL	CD19	Recruiting	Institut d’Investigacions Biomèdiques August Pi i Sunyer (Responsible Party)
NCT05149391	Phase 1	B Cell NHL	CD19 and CD20	Recruiting	Peking University Cancer Hospital & Institute
NCT05312476	phase II	R/ B-cell NHL	Igβ	Recruiting	The First Affiliated Hospital of Soochow University
NCT05260957	phase II	Aggressive NHL R/R NHL	CD19	Recruiting	University of Miami
NCT04089215	phase II	R/R NHL	CD19	Recruiting	Shanghai Ming Ju Biotechnology Co., Ltd.
NCT05420493	Phase 1	R/R NHL	CD19	Recruiting	Chongqing Precision Biotech Co., Ltd
NCT05757219	phase II	DLBCL	CD19	Recruiting	H. Lee Moffitt Cancer Center and Research Institute
NCT03758417	phase II	MM	BCMA	Recruiting	Nanjing Legend Biotech Co.
NCT05577000	Phase 1	R/R MM	BCMA	Recruiting	University of California, San Francisco
NCT04155749	Phase 1	R/R MM	BCMA	Recruiting	Arcellx, Inc.
NCT03943472	Early Phase 1	R/R MM	BCMA	Recruiting	Hrain Biotechnology Co., Ltd.
NCT04272151	Phase I/​II	R/R MM	BCMA	Recruiting	Chongqing Precision Biotech Co., Ltd
NCT05457010	Phase 1	AML, MDS	sparX	Recruiting	Arcellx, Inc.
NCT04219163	Phase 1	R/R AML	CLL-1	Recruiting	Baylor College of Medicine
NCT04835519	Phase I/​II	R/R AML	CD33	Recruiting	Beijing Boren Hospital
NCT04803929	Early Phase 1	R/​R AML(M4/​M5)	ILT3	Recruiting	Carbiogene Therapeutics Co. Ltd.
NCT05023707	Phase I/​II	AML	FLT3	Recruiting	The First Affiliated Hospital of Soochow University
NCT05488132	Phase I/​II	AML	siglec-6	Recruiting	Xuzhou Medical University
NCT04351022	Phase I/​II	AML	CD38	Recruiting	The First Affiliated Hospital of Soochow University
NCT05266950	Phase 1	R/​R AML	CI-135	Recruiting	Beijing Boren Hospital

Note: Data collection from https://clinicaltrials.gov/;DLBCL - Diffuse Large B-Cell Lymphoma, MM-Multiple Myeloma, AML-Acute Myeloid Leukemia, MDS-Myelodysplastic Syndromes, R/R - Relapsed or Refractory, ALL-Acute Lymphoblastic Leukemia, NHL-Non-Hodgkin Lymphoma, sparX- soluble protein antigen-receptor x-linker

**Table 3:** Exploring Resistance Mechanisms and Strategies in Chimeric Antigen Receptor T-cell Therapy for Hematologic Malignancies

Classification	Resistance mechanisms	Strategies
T-Cell related factors	•Lack of multi-cytokine producing Cells •Poor quality of donor T cells •Anti-CD19 scFv derived from murine •Inappropriate percentage of CD4 + and CD8 + CAR-T-cells •Nature of the costimulatory	•Early referral and leukapheresis to optimize the quality of T cells •Optimizing CAR-T cell design by incorporating additional co-stimulatory molecules •Using fully human or humanized scFvs instead of murine-derived ones to reduce immunogenicity •CRISPR/Cas9-engineered universal CAR-T cells •Multi-targeted CAR-T cells that target multiple antigens on tumor cells to overcome antigen loss or downregulation •Armored CAR-T cells are engineered to secrete cytokines or other therapeutic agents that enhance their activity in the tumor microenvironment •Combination therapy using CAR-T cells together with other treatments such as checkpoint inhibitors or chemotherapy to augment the immune response
Tumor Cell-Related Factors	•Tumor Heterogeneity •Antigen loss or down regulation •Lineage switching •Tumor Gene Mutation	
Tumor Microenvironment Related Factors	•Immunosuppressive chemokine signals and chemotaxis •Immunosuppressive cells such as Treg cells, MDSCs, and TAMs •Metabolic Fuel Deprivation	

### Acute lymphoblastic leukemia (ALL)

ALL is often fatal, with a dismal prognosis in r/r cases, particularly older patients [49]. The introduction of CAR-T cell treatment has transformed the clinical management of r/r B-cell ALL. Thus, despite significant progress, this disease remains an insurmountable obstacle for a subset of ALL patients.

The majority of ALL CAR-T cell treatments target CD19. According to Hay et al., the rates of CD19-negative relapse were 27% and 68%, respectively, lower than those of CD19-positive relapse [50]. Reduced persistence of CAR-T cells contributes significantly to CD19-positive relapse. Thus, enhancement of proliferation is crucial for preventing CD19-positive relapse. Clinical studies have shown that murine scFvs are more antigenic, resulting in CAR-T cell depletion [51]. In order to reduce the likelihood of immune reactions against CAR therapies, a viable approach would be to utilize fully human or humanized scFvs instead of those obtained from mice [51–53].

A novel approach involving CRISPR editing was undertaken to develop dual-targeted CD19/CD22 CAR-T cells for r/r ALL [54–56]. This approach confers benefits compared to autologous CAR-T cells, such as the ability to target two distinct antigens simultaneously, enhanced tumor detection and eradication capabilities, and a straightforward, rapid, and cost-efficient preparation procedure. The researchers, led by Yongxian Hu, discovered that CRISPR/Cas9 technology might be used to achieve precise and efficient gene editing, as well as the production of universal CAR-T cells [57]. Six patients received CTA101 infusion at doses of 1 (three patients) and 3 (three patients) X106 CAR -T cells/kg body weight, respectively. Currently, no dose-limiting toxicity, GVHD, neurotoxicity, or adverse events associated with genome editing have been observed. On the 28th day following the CTA101 injection, the complete remission (CR) rate was 83.3%. It is thus clear that these dual-targeted cells are better than single-targeted cells for treating r/r ALL [58].

Conversely, CD19-negative relapses were found to occur at an earlier stage in patients who had detectable CAR transgenes in their bloodstream within six months post-CAR-T cell treatment [50]. There is a 58‒68% antigen-negative relapse rate in B-ALL after CAR-T cell treatment [12, 59]. Studies have discovered that CD19-negative relapses occur as a result of lineage flipping. Pan et al. conducted a study on CD19 CAR-T cells and found a correlation between TP53 mutations and CD19-negative relapse in children diagnosed with B-cell ALL [60]. Additionally, Grupp et al. observed that a previously existing subset of CD19-negative tumor cell clones transformed into dominant clones following treatment with CD19 CAR-T cells, leading to CD19-negative relapse [61]. This recurrence can be explained in terms of natural selection [62].

In a mouse model, the application of CD7-CAR T cells as an immunotherapeutic intervention for T-cell ALL and other blood malignancies demonstrated significant anti-tumor efficacy in the presence of persistent antigen exposure [63]. These cells were also found to be more effective than their counterparts [63]. These findings demonstrate that CAR CD7-T cells may hold promise as a cellular immunotherapy for T-ALL.

In summary, both CAR-T cell persistence and specific function are crucial for preventing antigen-positive relapse, while mutations in antigens contribute to antigen-negative relapse. Further investigation is required to identify ways of extending the duration of treatment and overcoming resistance mechanisms to improve the survival rates of ALL patients.

### B-cell lymphoma

Non-Hodgkin’s B-cell lymphoma (NHL) encompasses a broad spectrum of diseases with diverse histological features, ranging from indolent to highly aggressive [64]. Anti-CD19 CAR-T cells represent an innovative treatment for B-cell lymphoma. However, although this treatment shows promise, about 60% of cases with refractory NHL respond poorly to the initial treatment, leading to subsequent relapse [10].

A recent clinical study introduced a new approach for the generation of gene-targeted CAR-T cells through the application of CRISPR-Cas9 technology [65]. By implementing a non-viral two-in-one strategy, researchers successfully incorporated PD-1 into the cells to enhance the immune function. The modified cells demonstrated superior efficacy in eradicating cancer cells in xenograft animal models. These findings suggest the potential of these integrated cells for NHL therapy. However, there is still the problem of recurrence, requiring further investigation. Therefore, an elucidation of the mechanisms underlying the failure of CAR-T cell therapy is necessary for improving outcomes.

Mechanisms contributing to failure include factors intrinsic to the tumor, mechanisms specific to CAR-T cells, and interactions between the cells and the host that lead to failure. In the ZUMA-1 clinical trial, biopsy specimens from 11 lymphoma cases with therapy failure were analyzed. Immunohistochemistry and/or flow cytometry showed that three of the 11 samples (27%) had lost the CD19 antigen [66]. In NHL, CD22 expression varies between aggressive and indolent tumor populations, ranging from 91 to 99%, respectively [67]. Hence, single-target therapy may be ineffective for treating NHL in cases of CD22 or CD20 under-expression or if tumor antigens are lost during treatment. To address this challenge, ongoing investigations are focusing on novel vectors capable of the simultaneous binding of several antigens, such as CD19/22 and/or CD19/20/22, on a single CAR molecule. In a study comparing two treatments targeting CD22 for recurrent B-cell hematological cancers, a novel IS7-CAR targeting the central epitope of CD22 showed superior efficacy [68]. This novel CAR showed higher affinity in vivo and effective treatment of lymphoma xenograft models, making it a potential alternative for refractory B-cell lymphoma [68].

Inherent failure mechanisms associated with CAR-T cells result in both poor expansion and functional impairment of the lymphocytes, ultimately resulting in adverse clinical outcomes. The reasons for failure may be linked to various factors, including suboptimal product quality in manufacturing or inferior quality of the donor T cells [69]. Therefore, exploring the options of optimal bridging treatment and assessing the use of radiotherapy and chemotherapy is imperative to ensure the availability of high-quality donor cells for the manufacture of CAR-T products.

The tumor microenvironment (TME) also contributes to treatment resistance in lymphoma. The interaction between immunosuppressive TMEs and CAR-T cells can reduce the proliferation and increase the depletion of CAR-T lymphocytes [70]. Previous reports suggest that the pre-treatment cancer burden influences CAR-T cell expansion and, thus, their therapeutic efficacy against tumors [71].

In conclusion, an understanding of the mechanisms responsible for the failure of the therapy in NHL is essential for developing rational strategies to address these difficulties. Novel vectors targeting numerous antigens in a single CAR, enhancing CAR-T cell persistence and function, and optimizing bridging therapies may help overcome the causes of therapy failure, ultimately improving outcomes for patients with B-cell lymphoma.

### Multiple myeloma

Despite recent advancements in multiple myeloma (MM) treatment, cases that do not respond to agents such as CD38-targeting antibodies and proteasomal inhibitors have a poor prognosis [72]. Thus, additional CAR-T cell treatments that can enhance anti-tumor effects with minimal toxicity are urgently needed.

An effective strategy for managing r/r MM involves the application of CAR-T cell targeting of B-cell maturation antigen (BCMA). Clinical research has shown that this approach can lead to profound and long-lasting remissions in patients [73]. Clinical trials assessing the efficacy of these cells have shown significant remissions during treatment of refractory MM and improvements in the patient quality of life scores [74–76].

However, single-target immunotherapies are subject to primary resistance and relapse. To address this issue, CAR-T cells that target both CD38 and BCMA have been designed. A clinical trial of these, including 23 patients, indicated that 20 patients (87%) achieved partial clinical remission with minimal residual disease negative, while 12 patients (52%) showed complete remission [77]. Furthermore, it has also been found that the targeting of both tumor cells and cancer-associated fibroblasts can enhance tumor clearing and extend survival in patients with refractory MM [78]. Animal models have also demonstrated improved sustained response rates when CART cells target both GPRC5D and BCMA [79].

GPRC5D exhibits highly specific expression in MM cells and can serve as a new target in MM patients who become resistant to BCMA-targeted therapy [80]. Incorporation of the GPRC5D-targeted scFv clone 109 with CAR-T cells resulted in the successful eradication of MM cells, even in animal models where the BCMA antigen escaped [80]. Phase I trials evaluating the therapeutic effect of these cells showed positive clinical treatment responses [81]. Additional investigation is needed to assess the effectiveness and safety of these cells in larger patient groups, explore potential combination therapies, and study the mechanisms by which GPRC5D expression is regulated in MM cells.

In order to decrease the immune response associated with mouse antigens, CAR-T cells that target entirely human BCMA have been created. These cells have shown notable rates of disease regression in patients who relapsed after receiving treatment with therapy that targeted mice antigens [82]. Furthermore, preliminary research has also shown that the combination with PD-1 inhibitors can promote the proliferation of CAR-T lymphocytes and increase their killing of MM cells [83].

Research suggests that BCMA expression may be preserved in myeloma patients who show disease progression on BCMA-targeted therapy, which may explain their response to subsequent different BCMA-targeted therapies [84]. Sequential use of various BCMA-targeted therapies has resulted in favorable outcomes in these patients [85, 86]. An analysis of the causes of resistance leading to relapse after this therapy has discovered an elevated ratio of monocytes/macrophages, a decrease in T-cell count, and an increased fraction of BCMA-positive plasma cells that express additional surface markers such as CD38, GPRC5D, and CD138 [86]. These findings lay the foundation for optimizing the performance of CAR-T cells that target BCMA.

In summary, the use of CAR-T cells is an emerging and feasible treatment for recurrent/refractory MM. However, further research is needed for the construction of optimally engineered cells to maximize the survival rate of MM patients and reduce treatment side effects.

### Myelodysplastic syndrome

Myelodysplastic syndrome (MDS) originates from clonal increases in either hematopoietic or pluripotent stem cells and is characterized by dysplasia, inefficient hematopoiesis, and peripheral blood cytopenia [87]. Although MDS patients receive standardized comprehensive treatment, their prognosis remains unfavorable. Currently, the effectiveness of CAR-T cell immunotherapy is being assessed in MDS patients.

Natural killer group 2 receptor (NKG2D) is a receptor specifically found on the surfaces of natural killer (NK) cells and certain T cells. The NKG2D ligand is expressed exclusively during pathological inflammatory states, making it a promising target for cellular immunotherapy. Given that MDS cells show significant overexpression of the NKG2D ligand, targeting these cells for treating MDS has been investigated [88]. However, a clinical trial in 2018 involving AML/MDS patients did not observe any objective tumor response following a single injection of low cell doses [89]. Although NKG2D-CAR-T cells could kill tumor cells in vitro, the researchers stated that certain components of the therapy might need to be modified and improved to maximize their anti-tumor-killing efficacy in clinical situations.

The safety of the NKG2D-CAR-T cell treatment CYAD-01 was recently assessed in a clinical trial, determining the recommended phase 2 dosing [90]. The multi-centre THINK study demonstrated good tolerability of CYAD-01 and promising anti-tumoral activity in refractory MDS, AML, and MM patients. These Phase I data provide supportive evidence for targeted NKG2D CAR-T cells for MDS.

CD123-targeted CAR-T cells have shown promise in differentiating high-risk MDS stem cells from normal progenitor cells in pre-clinical models, offering a potential approach for treating MDS [91]. However, there is currently insufficient clinical evidence on the efficacy of these treatments for MDS. The utility of autologous cells to treat MDS remains questionable. Promising treatment routes involve the targeting of numerous MDS-associated antigens, such as compound CAR-T cells (cCARs) and CAR-NK cell therapies. However, further study is required to confirm their therapeutic efficacy.

### Acute myeloid leukemia

The clonal expansion of primitive myeloid cells causes acute myeloid leukemia (AML). The disease shows high heterogeneity and is associated, in some cases, with poor prognosis. Although CAR-T cells are remarkably productive for treating B-cell tumors, their use for other malignancies, such as AML, presents difficulties. A significant challenge is the identification of suitable targets, as malignant myeloid cells share surface antigens with normal hematopoietic stem cells [92]. This shared expression can lead to long-term myelotoxicity when tested in clinical trials. Notably, both AML and normal bone marrow hematopoietic stem cells have high amounts of CD33 and CD123, which makes targeted CD123-CART cell therapy difficult because it may cause myelosuppression [93–95].

Preclinical research in animals and in vitro has demonstrated encouraging outcomes in addressing this problem by employing a fast-switching universal anti-CD123 CAR-T cell to eradicate CD123 + leukemia cells [96, 97]. A CD123-targeting module (TM123) is utilized to control the cytolytic reaction and cytokine release, which helps regulate the cytotoxicity of the product and reduce the toxicity of myelosuppression [96].

The effectiveness of this rapidly converted universal anti-CD123 CAR-T cell therapy was assessed in r/r AML. Three patients were enrolled, and the findings demonstrated clinical responses in all treated subjects; two cases achieved complete responses with incomplete hematological recovery, while one case showed a partial response [98]. Additionally, the targeting of CD38, found on most AML blast cells, has been found effective in AML cases that relapse after allogeneic hematopoietic stem cell transplantation (HSCT) [99]. CD70, CD22, and CD7 also represent potential targets due to their selective expression on AML blasts [100–102]. Nevertheless, despite encouraging in vitro outcomes, recent pre-clinical studies have demonstrated that CD70-targeted CAR-T cells were unable to eradicate leukemia in vivo [103].

Another promising target in AML is Siglec-6, a surface receptor explicitly found on both primary AML blasts and cell lines but not on normal hematopoietic stem cells. Targeting of Siglec-6 resulted in complete remission in mouse xenograft models [104]. Furthermore, approximately 35% of AML patients exhibit mutations in the oncogene nucleophosmin (NPM1c). Pre-clinical research suggests that CAR-T cells targeting NPM1c are strongly cytotoxic to leukemia cells and primary AML blasts, highlighting their potential clinical value for AML treatment [105].

In conclusion, finding targets for CAR-T cell treatment of AML remains challenging due to the sharing of surface-associated antigens between normal hematopoietic stem cells and malignant myeloid cells. Nevertheless, promising targets such as CD123, CD38, CD70, Siglec-6, and NPM1c provide potential avenues for the effective treatment of AML with reduced toxicities. However, the clinical efficacy of these requires verification in clinical studies.

## Mechanisms of resistance/recurrence associated with CAR-T cells in blood malignancies

The efficacy of CAR-T cell treatment for hematological malignancies is influenced by various factors, including the techniques used for CAR-T construction, proliferation and drug resistance in cancer cells, and TME immune suppression. It is essential to comprehend the influence of these elements on long-term therapeutic results.

**Fig. 2:**
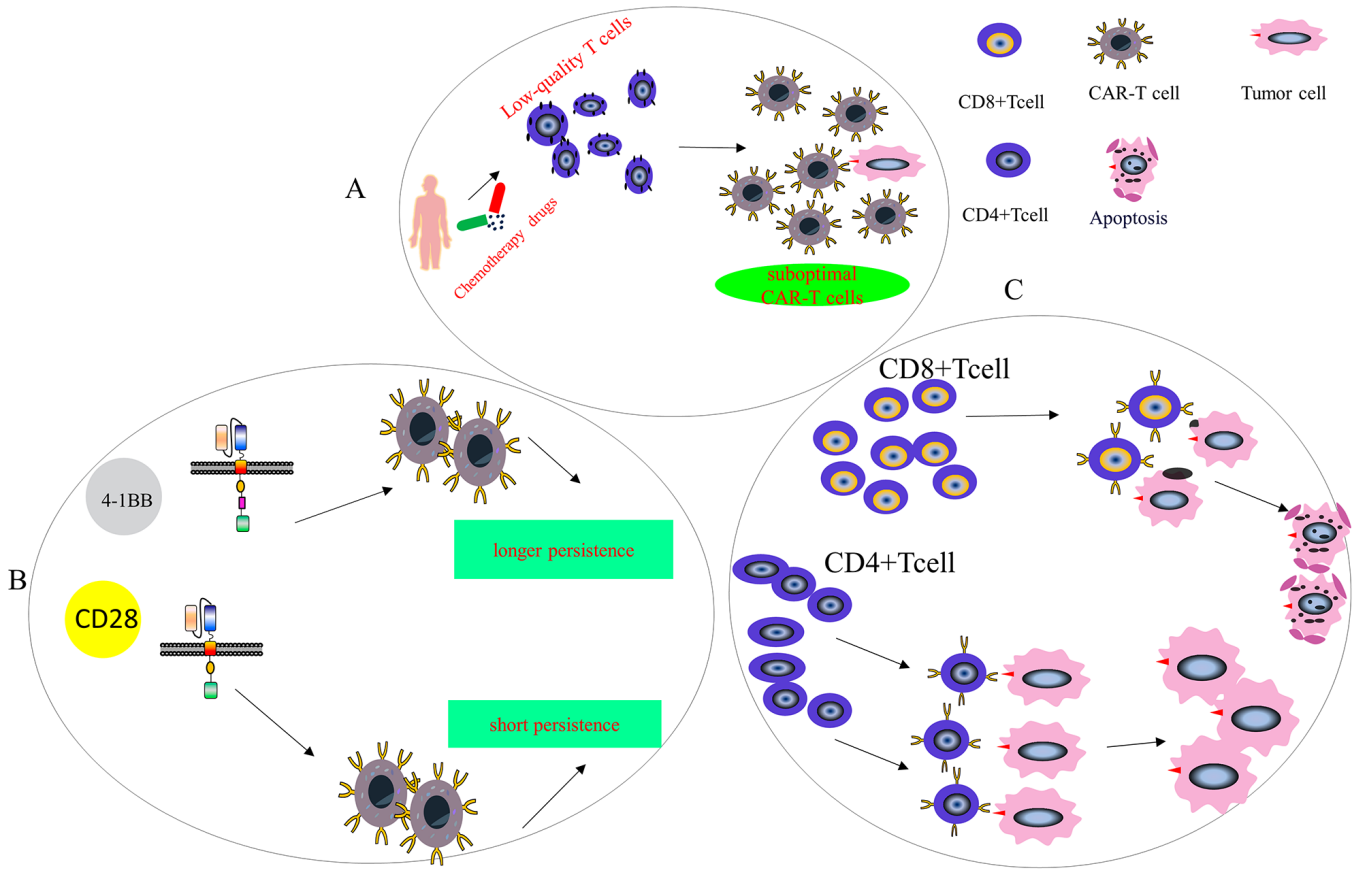
Antigen-positive relapse mechanism. **A**: T Cell Quality: Patients receiving chemotherapy drugs prior to T cell collection lead to low-quality T cells, resulting in suboptimal CAR-T cell products. **B**: Co-Stimulatory Domain: CART cells with 4-1BB co-stimulatory molecules persist in the bloodstream for a prolonged period than CART cells that structurally contain a CD28 co-stimulatory molecule. **C**: Different T Cell Subsets: CAR-T cells constructed by CD8 + T cells have a stronger tumor-killing ability than CD4 + CAR-T cells

**Fig. 3:**
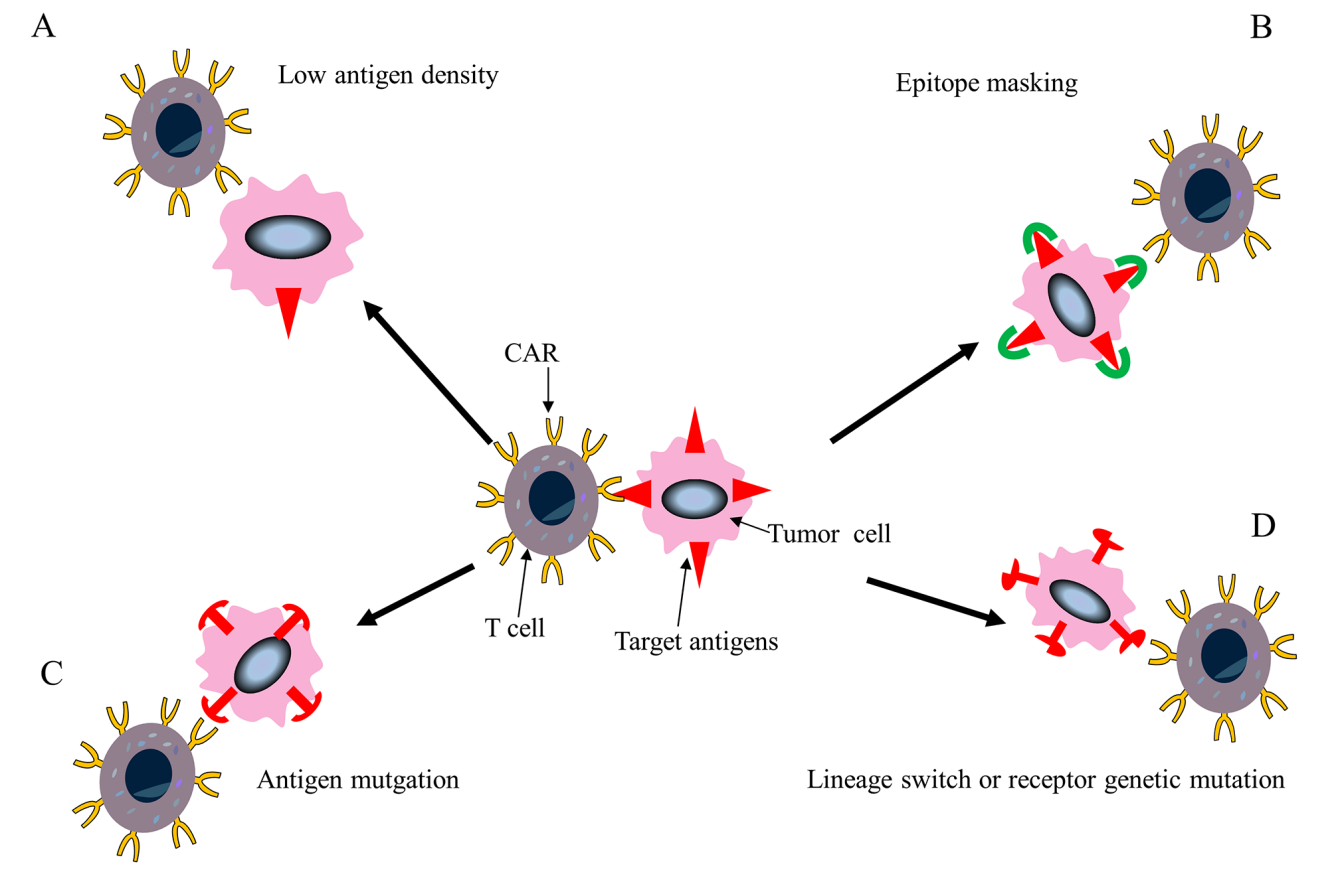
Antigen-negative relapse mechanism. **A.** Tumor cells downregulate surface target antigen to levels below the activation threshold of CAR T cells. **B**. Lentiviral modification of single leukemic cell for antigen expression, masking antigen epitope from CAR T cells. **C**. Tumor heterogeneity leads to antigen mutation. **D**. Tumor cells lineage switch or receptor genetic mutations leading to lack of extracellular epitopes recognized by CAR T cells

Despite the fact that CAR-T cells show promising results in ALL, there is a harsh reality to face, namely, that the relapse rate within one year post-infusion is approximately 50% [12]. Disease relapse shows two types of pattern, namely, early antigen-positive relapse (Fig. 2) and late antigen-negative relapse (Fig. 3) [106]. In-depth knowledge of the mechanisms underlying drug resistance and relapse in CAR-T cell therapy for blood cancers is crucial for maximizing treatment outcomes.

### Antigen-positive relapse

There is a close relationship between antigen-positive relapse and the presence of CAR-T cells in the body. Reduced CAR-T cell numbers have been observed in ALL patients, resulting in incomplete elimination of antigen-positive tumors [1]. While the factors that influence persistence are not fully understood, potential associations have been found between the functions and properties of co-stimulatory domains in the CAR, the initial T cell phenotype (CD4 + versus CD8+), and inherent T cell quality [107, 108].

#### T cell quality

High-quality T cells are essential for the successful production of CAR-T cells. Many patients suffer from lymphopenia after previous therapy, leading to insufficient numbers of T cells. Moreover, the use of clofarabine or doxorubicin chemotherapy is associated with inadequate or suboptimal CAR-T cell products [109]. Additionally, multiple prior chemotherapies can adversely affect T cell metabolic pathways in vivo, reducing the effectiveness and persistence of CAR-T cells [110].

#### Co-stimulatory domain

Clinical trials of tisagenlecleucel have revealed that CAR-T cells, including the co-stimulatory 4-1BB, persist for longer in the bloodstream than those containing CD28 co-stimulatory molecules [12]. This difference in persistence may be attributed to the importance of 4-1BB in T-cell activation and proliferation, extending T-cell survival and inhibiting immune escape [111]. Ongoing studies are focusing on the optimization of CAR-T cell persistence by incorporating a tyrosine-associated activation motif in the CAR [112]. Additional methods, such as numerous co-stimulatory domains or domains in various combinations, have also been examined in clinical and pre-clinical experiments.

#### T cell subsets affect the quality of CART Cell products

CD4 + CAR-T cells are not as effective in killing tumor cells relative to CD8 + cells due to decreased levels of intracellular perforin and granzyme [113]. Consequently, CD4 + CAR-T cells may require the recruitment of additional effector cells for effective elimination. Therefore, the selection of T cell subsets (including the CD4+/CD8 + ratio) CAR-T cell production is crucial for the successful treatment of blood diseases [107].

### Antigen-negative relapse

There are several potential causes of antigen-negative relapse in CAR-T cell treatment of blood malignancies (Fig. 3). The specific mechanisms of Antigen loss or modulation, Lineage switch, Tumor Heterogeneity, and Tumor Genetic Mutations in Antigen-negative relapse will be detailed below.

#### Antigen loss or modulation

Antigen loss or modulation (Fig. 3C) occurs when tumor cells cease producing the targeted antigen. This often results from alternative splicing, leading to the generation of CD19 isoforms [114]. Although genetic mutations affecting CD19 protein expression contribute to antigen-negative relapse, nearly half of such relapses are the result of alternative splicing reducing CD19 expression on the cell surface [114, 115].

In a study [116], it was found that tumor cells employ trogocytosis to acquire CAR molecules from CAR-T cells. This mechanism leads to short-term antigen loss and antigen masking(Figure 3B), ultimately resulting in CAR-T cell dysfunction. This mechanism primarily depends on antigen density and CAR sensitivity and is associated with tumor cell cholesterol metabolism. This form of intercellular communication operates regardless of CAR downstream signaling, the condition of CAR-T cells, the target antigen, or the type of tumor cell. However, it primarily relies on antigen density and CAR sensitivity and is linked to the cholesterol metabolism of tumor cells. Adjusting the administration of CAR-T cells to match individualized CAR sensitivities based on antigen density can partially reduce the transfer of CAR molecules induced by trogocytosis [116].

Previous targeted immune therapies have been observed to induce alterations in the phenotype of the tumor cell surface, allowing the cells to evade immune recognition and leading to antigen-negative recurrence. For example, blinatumomab, an anti-CD19 therapy used in ALL treatment, has been associated with CD19-negative relapse in clinical practice [117]. Similarly, anti-CD22 drugs can reduce the levels of surface CD22, thus allowing immune escape [118]. These therapies may reduce treatment efficacy when CAR-T cells target identical antigens, increasing the risk of antigen-negative relapse. It is worth mentioning that in the clinical trials studying tisagenlecleucel, prior treatment with blinatumomab was an exclusion criterion [12].

#### Lineage switch

Lineage switch (Fig. 3D) refers to the process in which leukemia cells undergo a transformation to a different lineage during relapse, accompanied by changes in the cell morphology and immunophenotype. The phenomenon of complete lineage conversion underlies drug resistance and recurrence in certain types of leukemias, thereby complicating subsequent disease diagnosis and treatment [119]. In the past, lineage changes were frequently seen in newborns before the development of targeted therapies [120]. However, recent reports have highlighted cases of myeloid lineage switch occurring after CD19 targeting in ALL cases with the ZNF384 fusion gene [121].

Anti-CD19-CAR-T cell treatment is linked with both partial and complete lineage switch as a treatment escape mechanism, which is induced by potential genetic oncogenic drivers [119, 122]. The exact role of lineage switch in actively evading CAR-T cell killing effects in other malignancies remains unclear. Nonetheless, understanding the mechanisms underlying resistance and relapse is essential for designing more advanced CAR-T cells.

#### Tumor heterogeneity

This review aims to explore the primary reasons and mechanisms responsible for the challenges in CAR-T cell treatment, focusing on how tumor heterogeneity, gene mutations, TME, chemokines, and metabolic fuels affect treatment efficacy. While antigen-negative relapse has its unique mechanisms, we will now delve deeper into the common mechanisms underlying resistance and relapse, explicitly examining the part played by tumor heterogeneity.

Tumors are inherently heterogeneous, which contributes significantly to antigen-negative relapse after treatment with CAR-T cells. The occurrence of antigen-negative relapse after treatment targeting CD19 highlights the diverse expression patterns of CD19 in B cells under specific conditions. Thus, it would be recommended to conduct a comprehensive analysis of CD19 expression prior to targeted CD19 treatment, as malignant cells in some patients may have reduced or no expression of CD19 [123]. Moreover, preexisting CD19 isoforms in malignant progenitor cells can potentially contribute to antigen-negative relapse after CD19-targeted therapy, especially in BCR-ABL1-positive ALL patients [124, 125]. Complete antigen loss may not be necessary to develop resistance to initially effective CAR-T cell therapy, even if reduced antigen expression is sufficient [126]. For example, despite over 90% of pre-B cells in ALL expressing CD22, the presence of CD22-negative sub-populations of ALL cells or cells with reduced can lead to resistance against CD22-targeted immunotherapy [127, 128]. These findings emphasize the significance of considering antigen density when designing effective CAR-T cells (Fig. 3A).

#### Tumor genetic mutations

Malignant cells are subjected to artificial selection pressure by CAR-T treatment, which may cause them to adapt. Due to this selection pressure, a tiny percentage of cancerous cells may acquire secondary genetic alterations that change antigen epitopes or cause antigen internalization, enabling them to elude identification by CAR-T cells [129].

Studies have demonstrated that tumor cells can evade targeted CD19 immunotherapy by the acquisition of frameshift mutations in exons 2 to 5 and point mutations in exon 3 of CD19, altering the levels of the antigen on the cell surfaces and thus evasion of the immune response [114, 130, 131]. Furthermore, it has been found that in MM, tumor cells can delete the BCMA gene, resulting in the evasion of immune attack by anti-BCMA CAR-T cells [132].

### TME

The TME influences both cancer growth and immune suppression and also impairs tumor cell lysis by CAR-T cells. The TME contains various cellular components that hinder CAR-T cell proliferation while favoring the survival of malignant cells. Abnormal chemotaxis, for instance, can promote the migration of malignant but not CAR-T cells. While the hypoxic and nutritionally deficient immunosuppressive environment benefits tumor cells, it reduces the proliferative and migratory functions, amongst others, of CAR-T cells. There is evidence that the TME contributes significantly to recurrence following CAR-T cell application [78, 133].

#### Immunosuppressive microenvironment

**Fig. 4:**
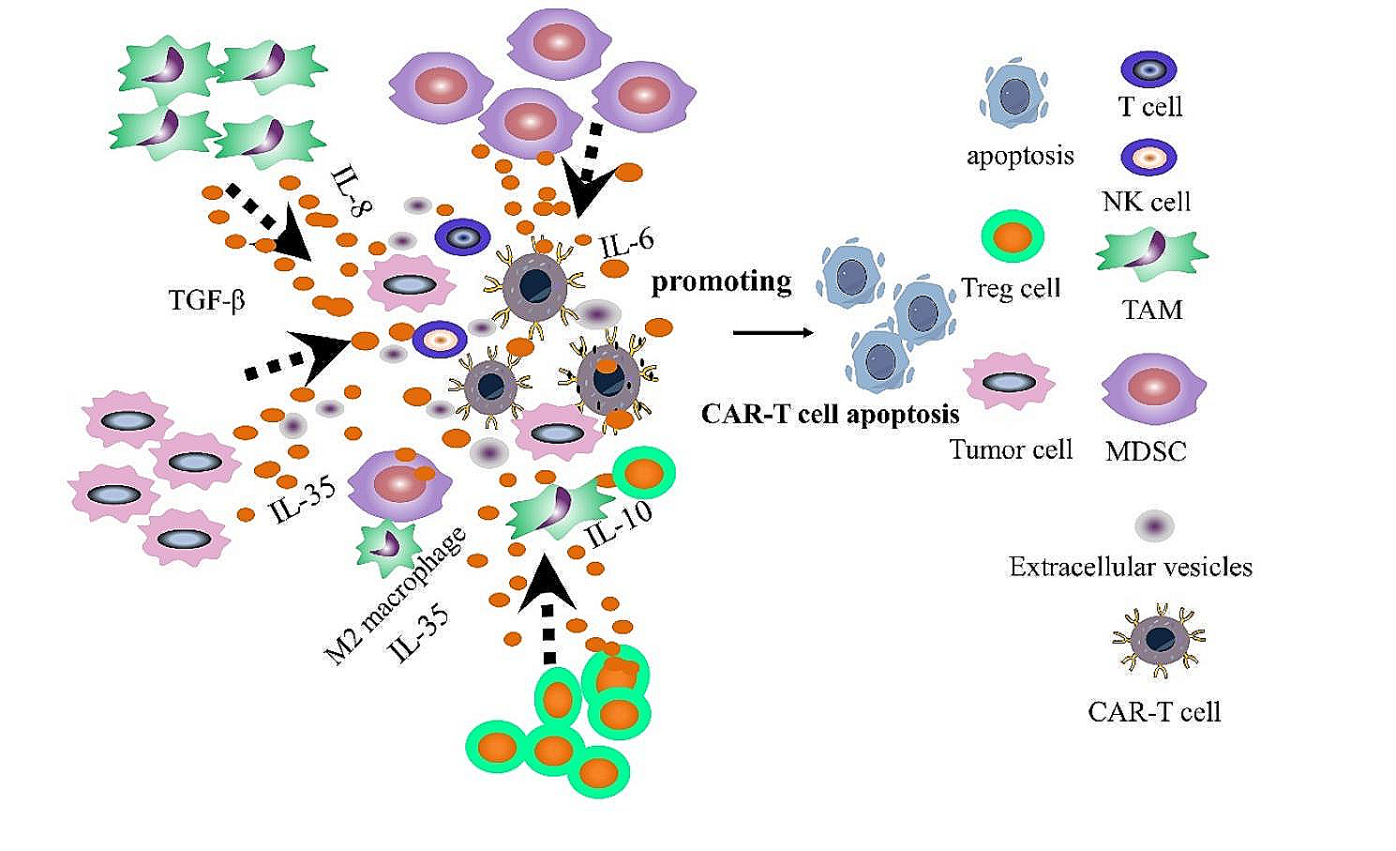
Tumor microenvironment. Immunosuppressive cells, including Treg, MDSCs, extracellular vesicles and TAMs, are present in the TME. They inhibit effector T cell function, enhance recruitment of Treg cells, and induce an immunosuppressive M2 phenotype by secreting cytokines such as IL-8, IL-6, IL-10, IL-35, and TGF-β.

The immunosuppressive TME exerts its inhibitory effects on CAR-T cells through various pathways involving abnormal cytokines, chemokines, and immunosuppressive cells such as myeloid-derived suppressor cells (MDSC), tumor-associated macrophages (TAM), and regulatory T cells (Treg cells) [16]. MDSCs directly target effector T cells, inhibiting their function, while TAMs impede T-cell function by the release of cytokines and amino acid-degrading enzymes. Furthermore, TAMs increase the recruitment of Treg cells and induce M2 macrophage polarization, further reducing anti-tumor activity (Fig. 4) [134–136]. Metabolites present in the TME, such as lactic acid, adversely affect both the survival and functioning of T and NK cells, promoting tumor immune escape [137–139]. Extracellular vesicles associated with the TME can also adversely affect CAR-T cells [140]. These characteristics of the TME provide potential therapeutic targets for optimizing immunotherapeutic agents. For instance, a pre-clinical investigation demonstrated that CD19-expressing extracellular vesicles enhanced the expansion and effectiveness of CD19-targeting CAR-T cells [141].

#### Chemokine signals and chemotaxis

A multitude of signaling molecules, including chemokines, mediate interactions between immune and non-immune cells within the TME. Chemokines are small chemotactic cytokines that attract specific cell types to their site of secretion. In malignant tumors, chemokine signaling and cytokines are essential components of the TME. Additionally, chemokines recruit effector cells, reshaping the tumor immune landscape.

Chemokines are secreted by tumor cells, immunocytes, and stromal cells, and their altered expression in the TME contributes to lymphocyte recruitment, tumor proliferation, and metastasis, together with inhibiting or promoting tumor growth [142]. For instance, activated cytotoxic CD8 + T cells and NK cells inhibit tumor growth by the release of effectors such as granzyme B and perforin. These cell types express a major chemokine receptor, and its ligands CXCL9 and CXCL10 recruit these cells into the tumor tissue [143]. Additionally, CD103 + dendritic cells are critical anti-tumorigenic chemokines, and the absence of CD103 + DCs can contribute to immune escape [144]. Tumor-associated macrophages (TAMs) release various cytokines, including IL-8, IL-6, and IL-10, which regulate the cell cycle and promote tumor growth [145]. Recent studies have reported that adoptive immunotherapy involving CAR-T cells overexpressing specific chemokine ligand receptors (CCL21, CCL19-IL7) enhances the recruitment of endogenous effector cells into tumor regions [146, 147].

#### Metabolic fuel deprivation

**Fig. 5:**
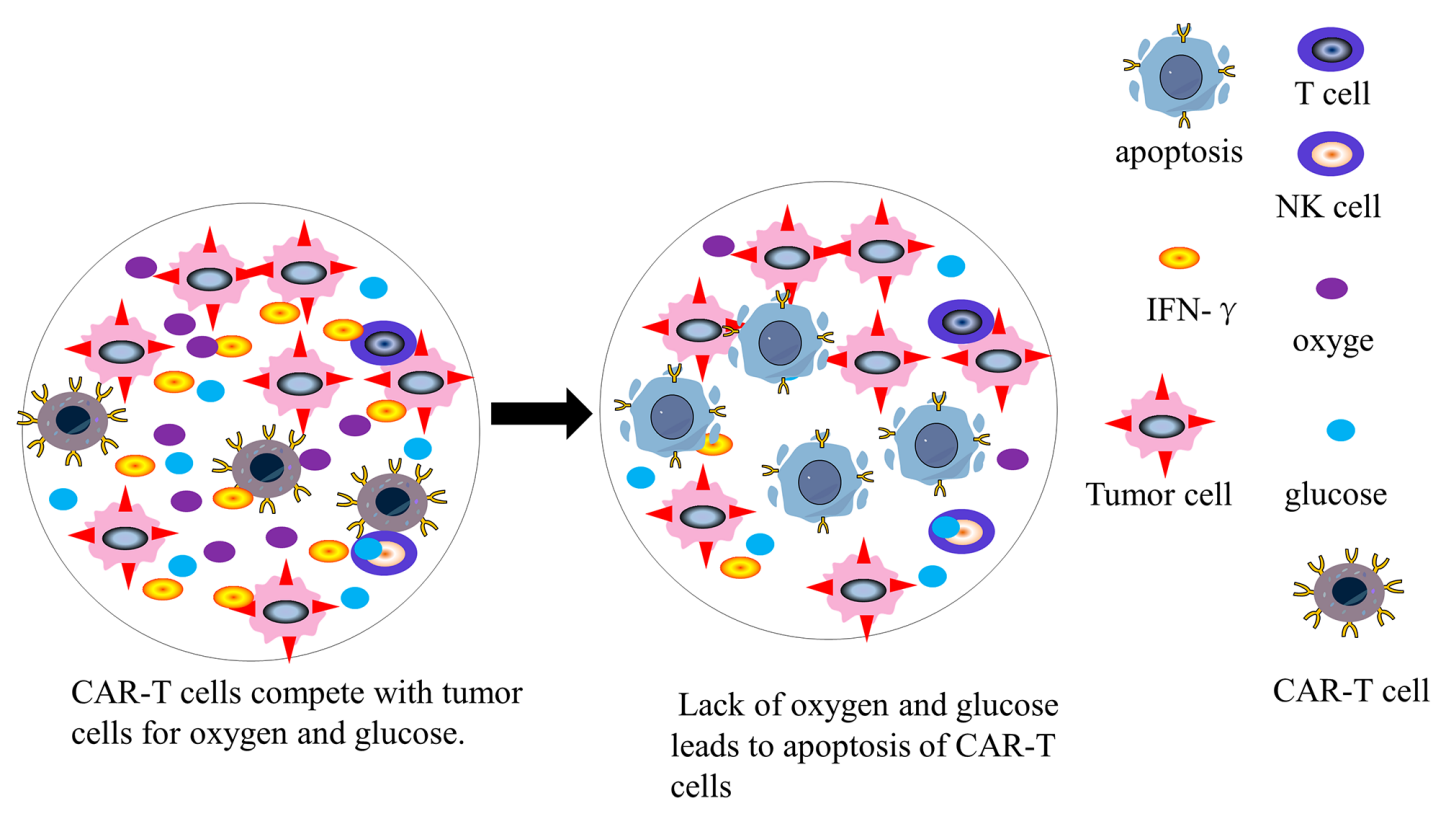
Metabolic fuel deprivation. The rapid growth of tumour cells consumes a large amount of glucose and oxygen, resulting in CAR-T cells lacking oxygen and glucose, which promotes cell apoptosis

The anti-tumor properties of CAR-T cells rely on major energy-producing processes, encompassing cell proliferation, tumor cell elimination, and cytokine secretion. To effectively combat tumors, CAR-T cells must compete with rapidly proliferating cancer cells for nutrients and oxygen. The promotion of glycolysis by T cell receptors (TCRs) is crucial for effector T cells to execute their functions and secrete anti-tumor cytokines such as interferon-γ (IFN-γ), enhancing their anti-tumor properties [148]. Both tumor and CAR-T cells utilize aerobic glycolysis to generate adenosine triphosphate (ATP) and sustain cellular activity. Unfortunately, overactive cancer cells in the TME consume excessive amounts of glucose, restricting glucose utilization by CAR-T cells, thus reducing ATP production and impairing their tumor-killing capacity (Fig. 5). A study demonstrated that excessive glucose consumption by tumor cells limits both the glycolytic capacity of T cells and IFN-γ production, ultimately contributing to tumor progression [149].

## Strategies to address the resistance (Fig. 6)

### Improving the quality of effector T cells

The quality of effector T cells is vital for tumor eradication and therapy efficacy. Precise detection and isolation of T cell subsets with robust activity for in vitro cultivation are necessary for optimizing clinical outcomes. While effector memory T cells are well-known for their cytotoxic and proliferative qualities, central memory T cells (TCM) have the benefit of generating immunological memory, which results in a longer-lasting ability to fight tumors. Research has demonstrated that in immunotherapies, TCMs are more persistent than effector T cells [150]. Additionally, stem cell memory T cells (TSCMs) can differentiate into progenitor cells and confer stem-like properties to CAR-T cells, addressing limitations such as poor in vivo persistence [151]. Therefore, the promotion of CAR-T cell differentiation into stem-like cells possessing the capacity for self-renewal and proliferation could serve as a viable approach to mitigate the risk of antigen-positive recurrence. Single-cell transcriptome analysis of PSC-derived cells has identified pathways for generating functional T cells, while updated PSC-to-iT platforms, including 3D artificial thymic organoid co-culture systems and gene editing technologies, offer potential for enhancing T-cell generation efficiency and functionality, advancing clinical applications from research to practical use [152].Additional research is necessary to comprehensively assess the characteristics of various effector T cells and their influence on CAR-T cell functions, with the aim of improving their use in clinical settings.

**Fig. 6:**
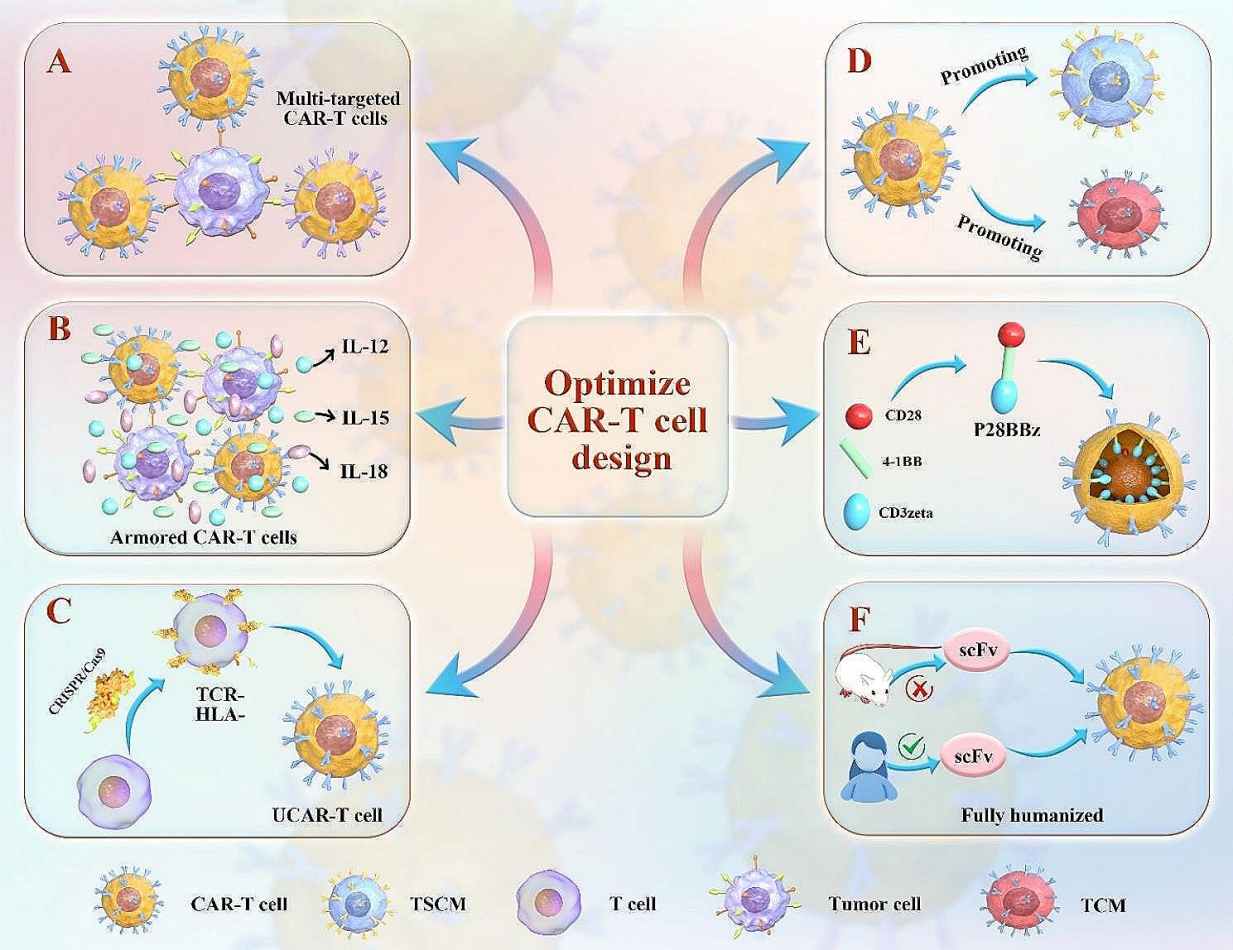
Optimization strategies for CAR-T cells. **A**: Multi-targeted CAR-T cells. **B**: Armored CAR-T cells. **C**: Universal CAR-T-Cells. **D**: Promoting the differentiation of CAR-T cells into central memory T cells (TCM) and stem cell memory T cells (TSCM). **E**: An appropriate co-stimulatory molecule. Three signaling domains of CD28, 4-1BB, and CD3zeta construct P28BBz receptor outperforms receptors containing only one or two of these domains. **F**: Humanized CAR-T cells

#### Selection of co-stimulatory molecules

Co-stimulatory molecules have a crucial role in the various functions of CAR-T cells, such as promoting T cell proliferation, enhancing their ability to persist, preventing apoptosis, and improving their ability to kill tumors. The successful demonstration of augmenting T-cell proliferation through the utilization of co-stimulatory signals has been accomplished by Renier Brentjens and Michel Sadelain [153, 154]. The co-stimulatory molecules that are currently being investigated in clinical and pre-clinical settings are 4-1BB, CD28, CD27, and ICOS. Distinct co-stimulatory molecules can enhance the generation of particular cytokines by CAR-T cells, wherein 4-1BB and CD28 facilitate the synthesis of IFN-γ and IL-2. The specific co-stimulatory domains selected during CAR design significantly affect CAR-T cell dynamics, including differentiation state, cytotoxic function, related toxicity, and anti-tumor response. Previous studies have shown that the P28BBz receptor, containing signaling domains of CD28, 4-1BB, and CD3zeta, outperforms receptors with only one or two of these domains in vivo, enhancing T-cell survival, cytokine release, and tumor-killing ability [155]. Recent investigations have revealed that combining 4-1BB co-stimulation with a refined variant of CD28 enhances T cell persistence, expansion, and resistance against exhaustion [156]. However, poor or inappropriate design may lead to CAR-T cell dysfunction [157]. Although there have been advancements, our comprehension of CAR signals remains limited, including intricate and interconnected signaling pathways. Additional investigation is required to clarify the complex connections between CAR-T cell signaling and enhance CAR design for more efficient and secure therapy.

#### Humanized CAR-T cells

CAR-T cells are recognized for their potential in treating hematological cancers, as they can selectively target and eliminate tumor cells. However, CARs incorporating non-humanized scFVs have raised concerns regarding immunogenicity and reduced efficacy, as shown in clinical trials. The inclusion of non-human scFvs can trigger the production of anti-CAR antibodies in humans, resulting in apoptosis of CAR-T cells and impaired lysis of cancer cells. In order to reduce immunogenicity and improve therapeutic outcomes, researchers have concentrated on creating fully humanized scFvs in CAR designs as a means of overcoming this obstacle.

Clinical trials investigating CAR-T cells with human-derived scFvs (Hu-CAR-T) have demonstrated several benefits relative to non-human-derived scFv. These advantages include reduced toxicity and immunogenicity, improved persistence, and favorable clinical responses [32, 158]. For example, one study constructed a CD19-targeted CAR that included a whole human scFv and removed any amino acid sequences that would be immunogenic from the CAR construct. It was discovered that using fully human scFv preserved clinical efficacy while significantly reducing immunogenic responses [159]. Another study evaluated a humanized scFv BCMA-CAR (CT053) for treating r/r MM, reporting an overall response rate (ORR) exceeding 80% without any observed immune-related adverse events [160]. Several reports have emphasized the importance of humanized antigen-recognition domains in overcoming immunogenicity [161, 162]. In summary, CAR-T cells based on fully humanized scFv exhibit low immunogenicity and can enhance the clinical efficacy of treating individuals with hematological malignancies.

#### Multi-targeted CAR-T cells

A significant problem is the loss of tumor-specific antigens during treatment, preventing recognition of the tumor. A feasible way of addressing this issue is the design of multi-targeted CAR-T cells. These may be constructed in different ways. Majzner and Mackall have outlined a number of strategies, such as producing distinct T-cell products separately and then infusing them simultaneously or sequentially [163]. Using two or more distinct CAR structures on T-cell surfaces is an additional strategy that increases CAR efficacy while lowering toxicity by allowing the targeting of several antigens on the tumor [163, 164]. These methods are currently being investigated for hematological tumors.

Pre-clinical studies, such as those conducted in glioblastoma, have demonstrated the potential benefits of using multiple CARs to prevent antigen escape [165]. Zah et al. designed a tandem CAR (CD19-CD20), finding that this could impede the progression of CD19-negative cancer cells in nude mice [166]. Similarly, Gill et al. reported that dual CAR-T cells (CD123 and CD19) could prevent antigen evasion on leukemic blasts following CD19-directed therapies [167]. Recent research has shown that using parallel CAR19/20 is more efficient in eliminating lymphoma cells in vivo compared to using single or tandem constructs [168]. Tong et al. designed tandem CARs that targeted CD19 and CD20, resulting in effective and long-lasting anti-tumor responses in individuals with r/r NHL [169]. The clinical efficacy of multi-targeted CAR-T cells is being examined in various trials. However, further exploration is necessary to determine their effectiveness and safety.

#### Armored CAR-T cells

The TME can suppress the CAR-T activity due to the presence of various cytokines. Therefore, the application of armored CAR-T cells has been suggested. The purpose of these altered cells is to produce extra proteins that increase the function of T-cells using second or third-generation CARs. The design concept is the production of cytokines or co-stimulatory molecules together with the CAR, aiming to enhance efficacy while reducing associated toxicity. Stimulatory cytokines have been employed for the enhancement of outcomes [170]. A subtype of armored cells, termed T-cells Redirected towards Universal Cytokine Killing (TRUCK), has been developed to enable site-specific cytokine production and secretion. TRUCKs can modulate the cytokine environment, resulting in the activation and enhancement of the killing capacity of both CAR-T cells and innate immune cells [171]. For example, IL-12 is involved in immune stimulation and the anti-tumor activity of NK cells. This process generates a positive feedback circuit of immune activation since activated NK cells release IFN-γ. A study has found that CAR-T cells secreting IL-12 are capable of targeting inaccessible lesions and immune cell recruitment. This finding indicates the advantage of using IL-12 [172].

Moreover, it has been observed that IL-15 can augment the cytotoxic characteristics of CD8 + T cells. This implies that CAR-T cells that secrete IL-15 could potentially boost the efficacy of tumor-cell eradication. CAR-T cells that secrete IL-15 exhibit a superior capacity to eliminate tumor cells compared to other cytokines [173–175].

To summarize, the utilization of armored CAR-T cells is advantageous in overcoming immune suppression within the TME. Adding cytokines and co-stimulators is a successful strategy for increasing the effectiveness of anti-tumor treatments while reducing any harmful side effects.

#### Universal CAR-T cells

Usually, autologous T cells are employed for the generation of CAR-T cells. Nevertheless, CAR-T cells derived from pluripotent stem cells or donors on a global scale have substantial benefits, including reduced expenses, a wider range of applications, and enhanced safety.

In contrast to autologous cells, universal cells can target antigens without requiring T-cell activation. To minimize immune rejection and graft-versus-host disease, genome editing, e.g., by CRISPR-Cas9, can disrupt TCR expression and HLA class I loci in donor T cells [176]. Additionally, the knockout of immune checkpoints such as PD-1 improves cytotoxicity [177, 178]. Two multi-centre clinical studies have evaluated universal anti-19 CAR-T cells (UCART19) in adult and pediatric cases r/r B-cell ALL, finding that while over 60% of cases achieved complete remission, side effects, including cytokine release syndrome, required monitoring [179].

UCART19 is a remarkable breakthrough in universal CAR-T cells and is beneficial for treating rapidly progressing diseases, eliminating the need to wait for autologous therapy. Currently, there are ongoing global pre-clinical and clinical investigations exploring the possibility of allogeneic immunotherapy [180, 181]. We are anxiously anticipating significant discoveries about universal CAR-T cells.

### Strategies for modulating the TME

#### Targeting suppressive cell types

**Fig. 7:**
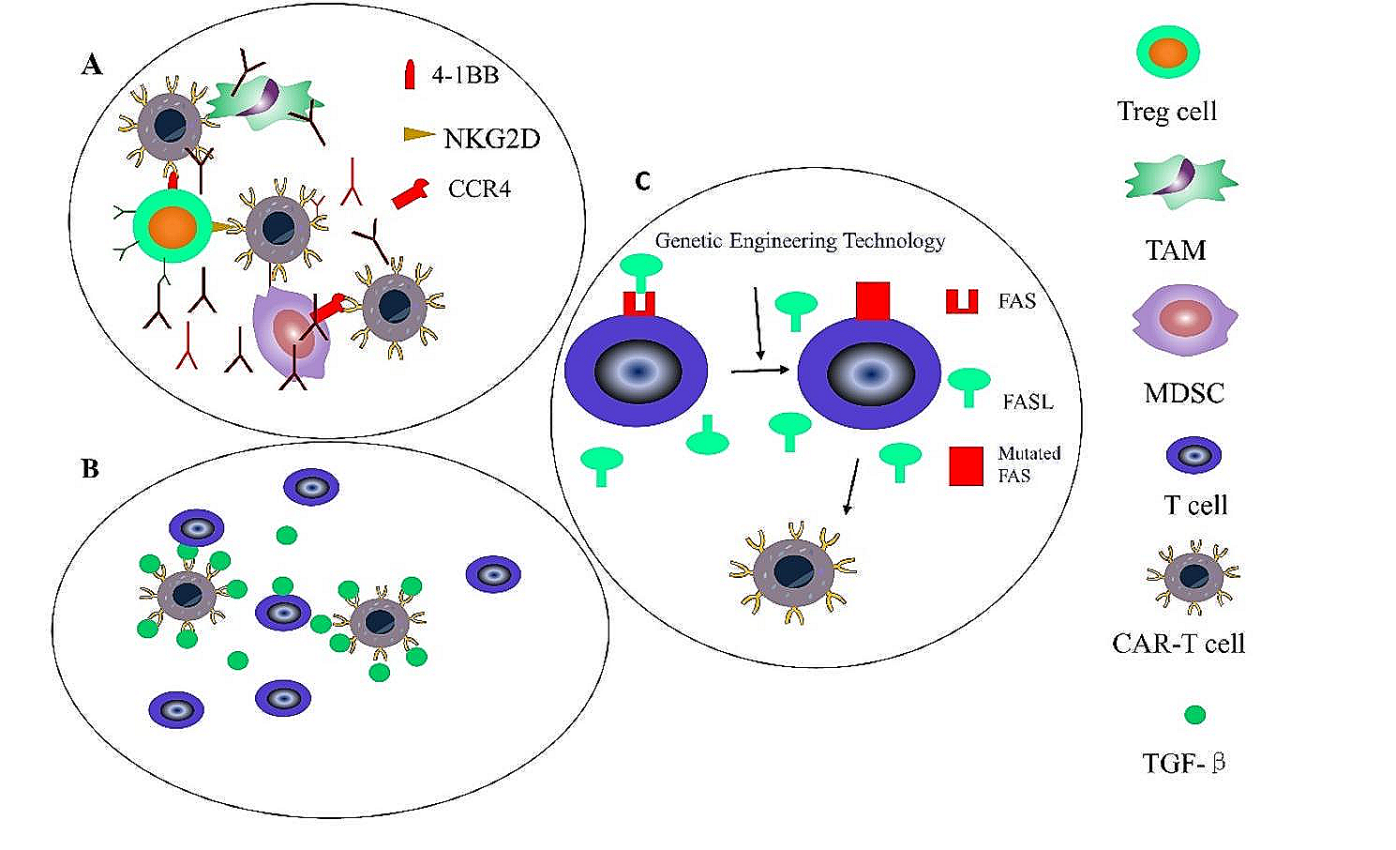
Strategies for modulating the Tumor Microenvironments. **A**: Targeting suppressive cell types. Using monoclonal antibodies and CAR-T cells to eliminate immunosuppressive cells such as T regulatory cells (Tregs), myeloid-derived suppressor cells (MDSCs), and tumor-associated macrophages (TAMs). **B**: Targeting suppressive cytokines. CAR-T cells targeting TGF-βinhibit the proliferative and anti-tumor abilities of cytotoxic cells. **C**: Targeting inhibitory signals. Genetic engineering technology that disrupts FAS signaling on T cells

One approach to mitigating immunosuppression in the TME involves the direct inhibition of suppressor cells in the TME. Tregs are a prime example of such inhibitory cells [182]. Targeted depletion of Tregs holds great potential as a viable and efficient approach to address this constraint. An option involves the combination of antibodies that target 4-1BB, a protein produced explicitly by Tregs that infiltrate tumors, with CAR-T cells that eliminate the Tregs. This approach enhances the treatment’s efficacy (Fig. 7) [183]. The use of a CAR that targets the C-C chemokine receptor 4 (CCR4) has also been proposed as a means of directly depleting Tregs to improve the TME [184]. Additionally, findings in animal models indicate that targeting IL18 can both reduce Treg numbers and enhance anti-tumoral activity [185].

In addition to Tregs, MDSCs and TAMs also exert inhibitory effects within the TME. Various approaches have been suggested to target these types of cells specifically. MDSCs that are linked to tumors have an increased expression of NKG2D, indicating a potential mechanism for mitigating immune suppression in the TME by MDSCs. In a xenograft TME model, NK cells carrying NKG2D-targeted CAR were used for the selective targeting of MDSCs, optimizing the anti-tumor activity of disialoganglioside (GD2)-targeted CAR-T cells [186]. Specific ablation of PD-1 on bone marrow progenitor cells also enhances immunity and inhibits tumor cell proliferation [187]. Another study demonstrated that the use of CRISPR/Cas9 technology to decrease the release of GM-CSF by CAR-T cells resulted in enhanced efficacy compared to cells with wild-type cells [188]. Thus, the targeting of inhibitory cells is effective for improving the effectiveness of CAR-T cell treatment.

#### Targeting inhibitory signals and suppressive cytokines in the TME

The intricate structure of the TME, which includes many cytokines and cells, effectively impedes the immune response against cancer. One potential technique to alleviate this immunosuppression is to target inhibitory cytokines and pathways. Notably, suppressive cytokines such as TGF-β, VEGF, IL-4, and IL-10 have been identified as key contributors to the immune evasion of tumors by reducing the immune response and preventing T-cell killing of the cancer cells (Fig. 7B).

In 2018, Andrew J Hou and his colleagues published research showing that the use of CAR-T cells to target TGF-β had a beneficial impact on tumor growth [189]. Moreover, FAS/Fas ligand (FASL) interaction in the TME reduces the effectiveness of CAR-T cells. Genetic engineering of the FAS gene is reported to prevent FASL-induced T-cell apoptosis and promote tumor killing by CAR-T cells [190]. However, further investigation is needed to determine whether genetic engineering that disrupts FAS signaling on T cells enhances the efficacy of CAR-T cell treatment.

Hence, additional investigation is required regarding the precise targeting of inhibitory signals within the TME. The identification of new targets for intervention, such as TGF-β and FAS signaling, could lead to the development of viable therapeutics. Understanding the mechanisms of immune suppression in the TME will offer significant knowledge and treatment approaches to counteract the immunosuppressive conditions in the TME.

### Combination therapy

**Fig. 8:**
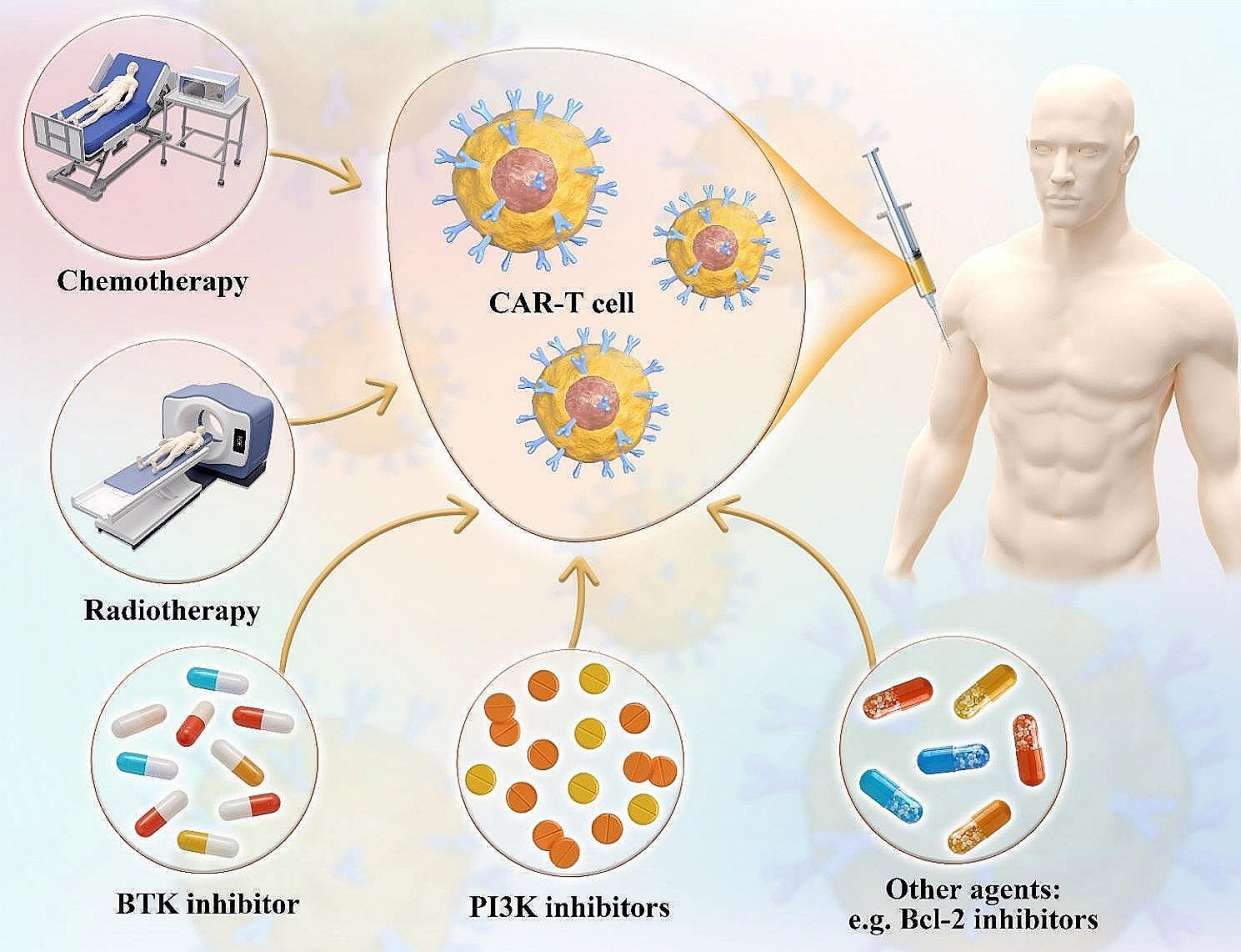
Combination therapy. Combination therapy using CART cells with chemotherapy drugs, radiotherapy, Bruton’s tyrosine kinase (BTK) inhibitors, PI3K inhibitors, and other agents such as Bcl-2 inhibitors enhance anti-tumor efficacy

Combining therapy can augment the efficacy of immune cell therapies. A study investigated the safety and efficacy of autologous stem-cell transplantation (ASCT) in combination with anti-CD30 chimeric antigen receptor (CAR30) T-cell infusion for relapsed/refractory CD30 + lymphoma. The results showed that this combined approach was well-tolerated and highly effective, leading to objective responses in all patients, including those with chemorefractory diseases, indicating promise for improving outcomes in this patient population [191]. Recent studies indicate that the combination of chemotherapy treatments with CAR-T cells can enhance the cells’ ability to multiply [192]. Lymphocyte depletion using a chemotherapy regimen (Fig. 8) containing Cyclophosphamide and Fludarabine promotes sustained proliferation of CAR-T cells, improving survival outcomes [192]. Moreover, the administration of lymphodepleting monoclonal antibodies prior to CAR-T therapy exhibits the potential to improve treatment results [192].

Radiotherapy, a standard treatment for lymphoma, can also promote an increased immune response by stimulating the production of antigens and cytotoxic T-cell recruitment [193]. While radiation is not typically employed for B-cell ALL, it has proven effective as a transitional treatment prior to CAR-T therapy in cases of extramedullary lesions in relapsed juvenile B-ALL [194]. The combined administration of radiotherapy and CAR-T cells warrants further exploration. However, optimal radiation dosing and target areas need to be determined through additional studies.

Bruton’s tyrosine kinase (BTK) inhibitors, such as the pioneering medicine ibrutinib, control the growth of tumors by regulating B cell proliferation and mortality. Co-administering CAR-T cells and BTK inhibitors in the treatment of lymphoma has the potential to decrease the likelihood of cancer returning and improve overall survival rates [195, 196]. Ibrutinib has been shown to reduce the immunosuppressive properties of chronic lymphocytic leukemia (CLL) cells and decrease the ratio of regulatory T cells to CD4 + T cells, thereby enhancing the efficacy of ibrutinib when combined with other immunotherapy methods [197]. Therefore, patients who have been previously treated with BTK inhibitors may exhibit a more favorable response to the infusion of CAR-T cells [198].

The overactivation of the PI3K pathway has been implicated in tumor growth. Targeting PI3K has been investigated in B-cell cancers [199]. PI3K inhibitors can augment the efficacy of CAR-T cells. The initial results of clinical research suggest that administering a PI3K inhibitor increased the length of time that patients with MM responded to treatment. The median duration of response was 17 months among the 72 participants [200]. Additional drugs, such as epigenetic modulators, gamma-secretase inhibitors, and Bcl-2 inhibitors, exhibit potential as options for augmenting the anti-tumor characteristics in CAR-T cells.

In summary, combination therapy is beneficial for improving the effectiveness of immune cell therapy. Further research endeavours should prioritize the identification of new targets and the optimization of current medicines in order to improve patient outcomes.

## Conclusion

In conclusion, an increased understanding of resistance associated with CAR-T cell treatment has generated new insights into this therapy. The main difficulties related to the management of blood cancers generally revolve around drug resistance and the recurrence of the disease. As a result, there is a worldwide emphasis on understanding these resistance mechanisms and devising techniques to overcome them while also addressing relapse concerns. Several novel strategies are now being assessed to address the issue of CAR-T resistance in blood cancers. Nevertheless, the efficacy of these therapy techniques remains unclear. Therefore, further research is required to enhance the effectiveness of the treatment and reduce the frequency of relapse.

## Data Availability

Not applicable.

## Abbreviations

FDA: Food and Drug Administration
DLBCL: Diffuse Large B-cell lymphoma
LBCL: Large B-cell lymphoma
scFv: Single-chain variable fragment
CARs: Chimeric antigen receptors
TAAs: Tumor-associated antigens
R/R AML: Relapsed/Refractory Acute Myeloid Leukemia
HSCT: Hematopoietic stem cell transplantation
TME: The tumor microenvironment
ALL: Acute lymphoblastic leukemia
BCMA: Homozygous B-cell maturation antigen
scFv: Single-chain variable fragments
PD-1: Programmed cell death protein 1
CLL: Chronic lymphocytic leukemia
